# Inflammatory Skin Diseases: Focus on the Role of Suppressors of Cytokine Signaling (SOCS) Proteins

**DOI:** 10.3390/cells13060505

**Published:** 2024-03-13

**Authors:** Antonia Cianciulli, Rosa Calvello, Chiara Porro, Dario Domenico Lofrumento, Maria Antonietta Panaro

**Affiliations:** 1 Department of Biosciences, Biotechnologies and Environment, University of Bari, I-70125 Bari, Italy; antonia.cianciulli@uniba.it (A.C.); rosa.calvello@uniba.it (R.C.); 2 Department of Clinical and Experimental Medicine, University of Foggia, I-71100 Foggia, Italy; chiara.porro@unifg.it; 3 Department of Biological and Environmental Sciences and Technologies, Section of Human Anatomy, University of Salento, I-73100 Lecce, Italy; dario.lofrumento@unisalento.it

**Keywords:** skin, inflammation, SOCS proteins, psoriasis, atopic dermatitis, JAK/STAT signaling pathway

## Abstract

Inflammatory skin diseases include a series of disorders characterized by a strong activation of the innate and adaptive immune system in which proinflammatory cytokines play a fundamental role in supporting inflammation. Skin inflammation is a complex process influenced by various factors, including genetic and environmental factors, characterized by the dysfunction of both immune and non-immune cells. Psoriasis (PS) and atopic dermatitis (AD) are the most common chronic inflammatory conditions of the skin whose pathogeneses are very complex and multifactorial. Both diseases are characterized by an immunological dysfunction involving a predominance of Th1 and Th17 cells in PS and of Th2 cells in AD. Suppressor of cytokine signaling (SOCS) proteins are intracellular proteins that control inflammatory responses by regulating various signaling pathways activated by proinflammatory cytokines. SOCS signaling is involved in the regulation and progression of inflammatory responses in skin-resident and non-resident immune cells, and recent data suggest that these negative modulators are dysregulated in inflammatory skin diseases such as PS and AD. This review focuses on the current understanding about the role of SOCS proteins in modulating the activity of inflammatory mediators implicated in the pathogenesis of inflammatory skin diseases such as PS and AD.

## 1. Introduction

The skin is a complex organ that forms a strong barrier against external influences and acts as a stage for various inflammatory processes, such as immune responses, against infection, tumor immunity, autoimmunity, and allergy. The skin barrier is, therefore, considered not only as a mechanical barrier able to limit water loss and prevent the entry of potentially dangerous micro-organisms and environmental elements but also as an active barrier that has the fundamental role of providing the first line of immunological defense against infections [1]. The outermost layer of the skin is represented by the epidermis, which is approximately 95% composed of keratinocytes, which are epithelial cells rich in structural proteins such as keratin and keratohyalin granules. Keratinocytes have multiple receptors on their membranes, including Toll-like receptors (TLRs), whose role is to detect the presence of molecules derived from micro-organisms. It was also reported that keratinocytes can also attract innate immune cells and release cytokines [2].

Immune system cytokines are known as soluble molecules that have a pivotal role in mediating the communication between immune cells but also between immune and non-immune cells, and some of these molecules are called interleukins (ILs). Their importance in the immune response was clarified in recent decades. Proinflammatory cytokines, such as tumor necrosis factor α (TNF-α), IL-6, IL-12, and IL-23, are, in fact, mainly secreted by macrophages and dendritic cells, and it is well known that they are able to activate the immune system by triggering and sustaining inflammation [3,4]. While cytokines normally contribute to host defense and immunological homeostasis, abnormal cytokines production leads to dysfunctional immune responses and immune-related diseases including autoimmune, rheumatologic, allergic, and inflammatory skin diseases [5]. Cytokines and eicosanoids are reported to play a pivotal role in the regulation of skin inflammation. Dermal and epidermal cell types constitutively produce various cytokines and eicosanoids, levels of which are regulated by physiological and pathophysiological events. Due to the complicated nature of bioregulatory positive and negative feedback mechanisms that exist, the significance and contribution of these inflammatory mediators to the overall pathophysiology of a skin lesion remain unclear [6]. Advances in understanding cytokine biology are rapidly being recognized as pharmacological opportunities. In this context, signals from cytokines are needed to mediate effective immune responses. In addition, in recent years, the importance of negative regulation by cytokine signaling in immunity has been highlighted, which, by suppressing excess immune responses, seems to be indispensable for maintaining the homeostasis of the immune system and of immune tolerance [7]. The signaling pathway regulated by cytokines is one of the cornerstones on which the immune system is based. Indeed, cytokines are involved in mediating the complex responses required to facilitate the proper differentiation of immune cells and all the functions that support robust, high-performance immunity [8]. Therefore, it is important that these signals are tightly regulated, and dysregulation supports immune system dysfunction, including excessive inflammation, and also promotes various immune-related pathologies. In this context, a special family of proteins known as suppressors of cytokine signaling (SOCS) is involved in the negative feedback regulation of cytokine signaling, ensuring its proper retention [9]. Cytokines play a crucial role in cell–cell communication because it is known that cells release these glycoproteins in response to typical developmental cues or environmental stimuli to interact with particular target cells that express the relevant receptor on their surface [10]. The engagement of a receptor starts a series of intracellular signaling cascades that result in appropriate cellular responses such as proliferation, differentiation, survival, and functional activation. The subsequent fading of receptor signaling is required to guarantee that the cell reaction does not become pathogenic. The SOCS proteins, therefore, represent one key mechanism through which this level of control is achieved [11]. In this manuscript, we will summarize recent advances in the SOCS proteins’ involvement in inflammatory-based skin disorders, and we will discuss their important role as regulatory molecules in two of the most common and best-characterized inflammatory skin diseases: atopic dermatitis (AD) and psoriasis (PS).

## 2. Skin Structure, Cytokines, and Skin Inflammation

The skin is the largest organ of the human body and serves as a physical barrier against external mechanical, biological, or chemical stimuli to safeguard internal organs and their physiological functions. It consists of three distinct and successive layers represented by the epidermis, dermis, and subcutaneous fatty tissue. The epidermis, or outer protective layer, is characterized by the presence of specific cells such as keratinocytes, Langerhans cells (LCs), and melanocytes. The dermis, the middle layer, which is abundant in immune cells, the extracellular matrix (ECM), fibroblasts, blood vessels, and skin appendages, is the layer specialized in giving the skin structure, nourishment, and support. The subcutaneous fatty tissue, a layer below the dermis that is adipocyte-rich, serves as an energy reserve further connecting the skin to the connective tissue below and supplying growth factors to the dermis [12].

The skin is, therefore, frequently exposed to various external stimuli such as UV radiation, contact with irritating substances, and repeated mechanical stimuli but also allergens and infections that can cause inflammation within its structure. Skin inflammation can also be caused by intrinsic factors such as autoimmune responses or hereditary mutations. Inflammatory phenomena that can affect the structure of the skin can be responsible for skin disorders linked precisely to inflammation. In this respect, it is known that inflammatory skin disorders are characterized by a powerful activation of both the innate and adaptive immune system. Immune system activation is responsible for increasing proinflammatory cytokines production, determining, especially in keratinocytes and fibroblasts, a shift in gene expression that leads to the wound-healing phenotype of these cells [13]. Different forms of skin inflammation can arise depending on the cytokine profile, the presence of particular immune cells, and the intensity of the response. Among the possible skin inflammatory diseases, PS is undoubtedly the most common inflammatory disease, affecting about 2–3% of the population. This inflammatory skin disease is a T-helper 17 (Th17)-mediated disease. It is characterized by severe chronic inflammation without a fibrous component in which the major cytokines involved, the interleukins IL-17, IL-22 and IL-23, are those that define the disorder, leading to epidermal hyperproliferation, abnormal differentiation of the keratinocytes, and barrier function disruption which manifests itself as red scaly patches forming on the skin. Patients affected by PS are exposed to increased risk for other serious and chronic health problems such as psoriatic arthritis as well as metabolic syndrome and cardiovascular diseases, which face increased morbidity and mortality [14,15]. In addition to PS, another inflammatory skin disease is AD, or eczema. This is a very frequent inflammatory disease characterized by signs such as redness, itching, and widespread barrier dysfunction. This inflammatory skin condition is characterized by the release of T-helper 2 (Th2) cytokines with strong upregulation of IL-4 and IL-13. In chronic eczema, instead, IL-17, IL-22, and interferon (IFN)-γ become more important [16].

Moreover, it is becoming increasingly evident that IL-1 family cytokines, as well as IL-6 cytokines, play a pivotal role in the pathogenesis of inflammatory disease at barrier sites [10,17,18]. Cytokines, therefore, are known to play important roles in many fundamental biological processes, including cytokine-induced signal transduction pathways, and it is clear that their excessive or prolonged release can be detrimental. It is therefore necessary to modulate the release of inflammatory cytokines at the level of the skin barrier in order to restore homeostasis in inflammatory skin disease [19,20]. All these observations underscore the importance of negative regulators of inflammatory cytokine release, such as the SOCS protein family, in the modulation of cytokine signaling. Therefore, the possibility of modulating the members of the SOCS protein family could become very important in the containment of inflammatory skin diseases such as PS and AD [21,22,23].

## 3. SOCS Protein Family

Members of the SOCS protein family are able to play an important role by acting as negative regulators of cytokine signaling by negatively regulating the Janus kinase/signal transducer and activator of transcription (JAK/STAT) signaling pathway [24]. The SOCS protein family, in particular, contains eight members that include SOCS1 to SOCS7 and cytokine-inducible SH2-containing protein (CIS), which are known to be implicated in many immunologic-related pathologies as well as inflammatory diseases including skin inflammatory diseases (Table 1). In this context, CIS, SOCS1, and SOCS3 are well characterized as molecules able to act as negative feedback regulators of the JAK/STAT pathway, whereas the biological functions of other members are less known and still under investigation [8,25].

Among all the SOCS family members, it is known that SOCS1–SOCS3 and CIS mostly regulate the JAK/STAT pathway, although SOCS4–SOCS7 seem more involved in acting on growth factor receptor signaling pathways. In any case, SOCS protein members generally counteract the activation of the JAK/STAT pathways by tagging target proteins for ubiquitination and consequent proteasomal degradation. This process proceeds by recruiting the E3 ubiquitin ligase scaffold via the interaction of the SOCS box motif with Cullin-5 (elongin B and elongin C). Because the SH2 domain defines the SOCS protein members’ binding processes, each SOCS family member has a unique binding mechanism [9,26,27]. The JAK/STAT pathway is activated by cytokines binding to cognate receptors, which, in turn, causes JAKs to phosphorylate tyrosine residues at the receptor level. As a result, STAT is attracted to the region where gene transcription takes place, and this step is followed by its dimerization and nuclear translocation [28].

Cytokines, including ILs and IFNs, interact with various extracellular ligands through their cell surface receptors, leading to the activation of intracellular JAK family kinases and STAT proteins [29]. In this context, SOCS proteins, characterized by a central SH2 domain and a conserved C-terminus, play a crucial role in regulating cytokine responses at various inflammatory sites. SOCS proteins are, in fact, able to strongly inhibit the JAK/STAT signaling pathway regulating Th1–Th2 cell balance and reducing Th2-induced inflammation. SOCS proteins expression modulation is able to influence the inflammatory cascade at different levels, such as Th2 cell differentiation, activation, and cytokine effects [23]. The importance of SOCS proteins in modulating the JAK/STAT pathway has been highlighted in recent years by various studies and, in this regard, this class of proteins is emerging as a possible new therapeutic target in the treatment of inflammation-related pathologies, including inflammatory skin diseases [30,31,32].

### 3.1. SOCS1 and SOCS3 Proteins

As previously reported, among the members of the SOCS protein family, SOCS1 and SOCS3 have been the most characterized for their role in the JAK/STAT signaling pathway. It is known that they play pivotal roles in cytokine regulation [21,33].

With regard to SOCS1, it is known that it is a 23.4 kDa protein mainly involved in the regulation of JAK/STAT and NF-κB pathways through ubiquitin-mediated proteasomal degradation. In particular, it down regulates IFN-γ-induced signaling pathways, as demonstrated in SOCS1 knockout mice, whereas SOCS3 is a 24.8 kDa protein involved in the negative regulation of proinflammatory cytokines expression, such as IL-6, and anti-inflammatory cytokines, such as IL-10, via selective binding to the gp130 receptor [34,35]. Despite having different activities, SOCS1 and SOCS3 have one thing in common with the other SOCS proteins members: a kinase inhibitory region (KIR) in the N-terminal domain that serves as a pseudo-substrate. Due to the presence of KIR upstream of the SH2 domain, SOCS1 and SOCS3 exhibit additional direct inhibitory mechanisms on JAK tyrosine kinase activity in addition to the general ubiquitylation and degradation activities [36]. In detail, SOCS1 can bind to the activation loop of JAK, preventing, in this way, the JAK activity and tyrosine phosphorylation of STAT1. In order to increase the inhibitory action on IFN signaling pathways, SOCS1 can also bind to the IFN-γ receptor directly, as well as to type I IFN receptors [37]. As opposed to that, the major kinase inhibitory effect of SOCS3 is caused by the interaction between the SOCS3 KIR and the JAK2 kinase domain, which in turn is determined by the lower affinity expressed by the SOCS3 SH2 domain for loop binding activated by JAK. Furthermore, SOCS3 can inhibit JAK/STAT signaling by interacting with a variety of cytokine receptors. For instance, JAK2 and STAT5 activation is inhibited by SOCS3 binding to the Tyr401 region of the erythropoietin receptor [38]. Moreover, SOCS3 interacts with other cytokine receptors including the leptin receptor, the gp130 receptor, the IL-12 receptor, and the granulocyte-colony-stimulating factor (G-CSF) receptor (namely Tyr085, Tyr 757, Tyr800, and Tyr729), exhibiting an interference very similar to the one shown in the JAK/STAT pathway [24,39].

### 3.2. Other SOCS Proteins

In addition to SOCS1 and SOCS3, the SOCS protein family also consists of other members. The inhibitory mechanisms of other SOCS proteins members, however, are far less studied. In this respect, CIS is a 28.6 kDa protein that is reported to be able to inhibit the STAT5 signaling induced by cytokines through substrate competition. In this case, the SH2 domain of the CIS competitively binds to tyrosine residues that are phosphorylated in activated cytokine receptors instead of JAK. SOCS2, like CIS, is a 22 kDa protein that uses competitive binding via the SOCS box to prevent growth-factor-mediated activation of STAT5 [40]. Only SOCS1 and SOCS3 have been shown to interact with JAK and decrease its activity directly through KIR [41]. Because SOCS2 lacks KIR, the probability of a direct interaction with JAK is minimal. Interestingly, recent research has demonstrated that SOCS2 may directly interact with JAK2 to adversely affect the JAK2/STAT5 signaling pathway, reducing the formation of natural killer cells [42]. Remarkably, SOCS2 is able to antagonize other SOCS members. For instance, SOCS2 can antagonize SOCS1 and SOCS3 into negatively regulating IL-2 and IL-3 signaling [42]. Moreover, SOCS4 and SOCS5 are proteins of 50.6 and 61.2 kDa, respectively, that are involved in the regulation of epidermal growth factor (EGF) signaling. The SH2 domain of SOCS4 and the N-terminal part of SOCS5 are thought to interact with the EGF receptor (EGFR) through a phosphorylation-dependent and a phosphorylation-independent interaction, respectively, and the docking of SOCS4 to EGFR submits the activated EGFR to proteasomal degradation through the recruitment of E3 ubiquitin ligase. In addition, SOCS4 has been reported to have the same binding affinity both to STAT3 and to EGFR, thus suggesting that SOCS4 is also effective in attenuating STAT3 activation [43,44,45].

In addition, it was reported that SOCS5 also targets proteins such as IL-6R, IL-4R, and EGFR for proteasomal degradation, allowing it to negatively regulate cytokine receptor signaling [46]. As a result, SOCS5 can contribute to Th1/Th2 differentiation by inhibiting IL4-dependent STAT6 activation [45].

Within the SOCS protein family, SOCS6, a 59.5 kDa protein, can also be included. The inhibitory mechanism of SOCS6, similarly to SOCS1, SOCS5, and CIS proteins, is likely carried out through ubiquitination and proteasomal degradation, in which E3 ubiquitin ligase is replaced with heme-oxidized IRP2 ubiquitin ligase1. SOCS6 is well recognized for its involvement in regulating the insulin signaling pathway through the inhibition of the insulin receptor substrate 1 (IRS1), extracellular signal-regulated kinases (ERK1/2), and protein kinase B. SOCS6, like SOCS2, can interact with other SOCS proteins; moreover, the N-terminal domain of SOCS6 enhances its nuclear localization which, in turn, regulates STAT3 [47].

Finally, SOCS7, a 62.9 kDa protein, is a well-known protein that inhibits insulin-like growth factor I receptor (IGFIR) signaling [48]. SOCS7 is able to modulate IGFI signaling by degrading IRS1 docking on the cytoplasmic domain of active IGFIR via the SOCS box; also, the interactions between the SH2 domain of SOCS7 and IRS2 and IRS4 inhibit downstream signaling. Through the interaction of JAK2/STAT3 and JAK2/STAT5, SOCS7 can alter the JAK/STAT signaling pathway generated by leptin and prolactin [48,49,50].

**Table 1:** SOCS protein family members’ action targets and cell signaling effects.

Name	Molecular Weight	Target	Effects on Cell Signaling	References
**SOCS1**	23.4 kDa	Ubiquitin-mediated proteasomal degradation	Regulation of JAK/STAT and NF-κB pathways Negative regulation of IFN-γ-induced signaling pathways	[34,35,36,37,41]
**SOCS2**	22.0 kDa	Competitive binding via the SOCS box	Prevents growth-factor-mediated activation of STAT5 Negative regulation of JAK2/STAT5 signaling pathway	[40,42]
**SOCS3**	24.8 kDa	Selective binding to the gp130 receptor	Negative regulation of proinflammatory cytokines expression	[24,34,35,36,38,39,41]
**SOCS4**	50.6 kDa	Epidermal growth factor receptor (EGFR)	Regulation of EGF signaling STAT3 signaling attenuation	[43,44,45]
**SOCS5**	61.2 kDa	Epidermal growth factor receptor (EGFR) IL-6R, IL-4R, and EGFR Proteasomal degradation	Negative regulation of IL-6, IL-4, and EGF release Negative regulation of STAT6 activation Activation of Th1/Th2 differentiation	[43,44,45,46]
**SOCS6**	59.5 kDa	Ubiquitination degradation Proteasomal degradation Insulin receptor substrate 1 (IRS1)	Extracellular signal-regulated kinases (ERK1/2) inhibition Protein kinase B inhibition Regulation of the insulin signaling pathway	[47]
**SOCS7**	62.9 kDa	Insulin-like growth factor I receptor (IGFIR) Insulin receptor substrate 2 (IRS2) Insulin receptor substrate 4 (IRS4) Interaction of JAK2/STAT3 Interaction of JAK2/STAT5	Regulation of the insulin signaling pathway JAK/STAT signaling pathway modulation	[48,49,50]

## 4. SOCS Proteins in Inflammatory Skin Diseases

**Figure 1:**
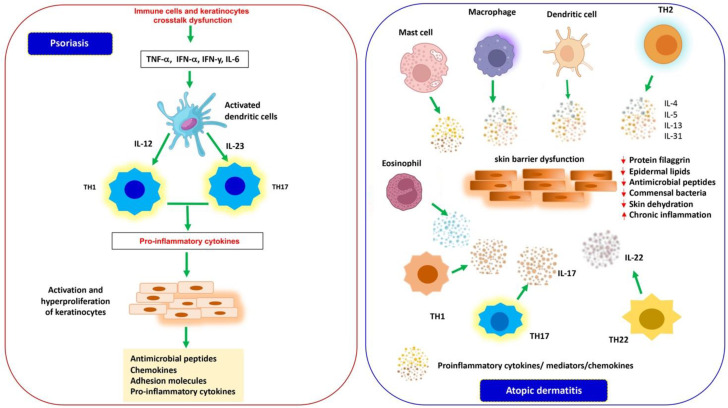
Inflammatory mediators implicated in the pathogenesis of PS and AD. In PS (**left**), a dysregulation of the crosstalk between cells of the innate and adaptive immune system and keratinocytes induces the production of proinflammatory cytokines that activate dendritic cells. Activated dendritic cells secrete cytokines (IL-12 and IL-23) that guide the differentiation and proliferation of Th-1 and Th-17 lymphocytes, in turn releasing a wide variety of inflammatory mediators and determining the hyperproliferation of keratinocytes with an increase in thickness of the epidermis and production of antimicrobial peptides, chemokines, and proinflammatory cytokines, contributing to the amplification of psoriatic inflammation. In AD (**right**), multiple factors cause a dysfunction of the innate and adaptive immune system of the skin barrier associated with a reduction in the structural protein filaggrin, epidermal lipids, and antimicrobial peptides and a dysbiosis of commensal bacteria determining a loss of epidermal integrity, dehydration, chronic inflammation of the skin, and increased sensitization to allergens and infections.

Many cytokines play key roles in skin inflammatory diseases through both immune and non-immune signaling pathways [51]. In mammals, the JAK/STAT pathway is implicated in several biological processes of the organism such as differentiation, cell proliferation, apoptosis, homeostasis, and immune regulation pathways [52]. It is the primary mechanism used by most cytokines for signal transduction and is regulated by different types of molecules, including SOCS proteins [53]. Several reports highlight that SOCS proteins are associated with inflammatory disorders and have an influence on cytokines, growth factors, and various types of cells such as keratinocytes and cells of the immune system involved in skin inflammation pathways [54,55]. Below, we discuss the current actions of SOCS proteins in modulating, through various signaling pathways, the activity of inflammatory mediators implicated in the pathogenesis of the most common forms of inflammatory skin diseases, such as PS and AD (Figure 1), as well as other skin inflammatory conditions, including wound healing.

### 4.1. SOCS Proteins in PS

PS represents a chronic, immune-mediated, and inflammatory disease of the skin, after relapsing, that affects 2–3% of the world’s population, varies by region, and can appear at any age [56,57,58]. It is a complex pathology whose etiology, to date, has not been completely clarified. It is believed that the combination of genetic, environmental (mechanical stress, alcohol, drugs, smoking, and infection), and immune factors leads to the onset and progression of the disease [57,59,60,61], negatively influencing the daily life of those who suffer from it. The individuals affected by this pathology, in addition, can often develop many other inflammatory based diseases such as psoriatic arthritis and cardiovascular and metabolic disorders [62,63,64,65,66,67,68,69].

The hallmarks of the disease are represented by well-circumscribed, red, erythematous papules and plaques covered with silvery scales caused by hyperproliferation and impaired differentiation of epidermal keratinocytes associated with inflammation of the epidermis and dermis [70,71]. The lesions can be asymptomatic or itchy and are located in most cases on the extensor surfaces of the elbows and knees, on the scalp and in the sacral region and can often cover the entire skin [54,72]. The histopathological characteristics of the disorder are determined by a quantitative alteration of the keratinocytes and their premature differentiation along the epidermal layers, which lead to a thickening of the epidermis, a parakeratosis, and inflammatory cell infiltration in psoriatic skin that is supported by increased dermal vascularization [72,73]. Several reports show that the pathogenesis of PS is determined by a dysregulated crosstalk between cells of the innate and adaptive immune system and keratinocytes. Psoriatic plaques are characterized by an increased number of dendritic cells, as well as of Th cell populations and an overexpression of proinflammatory cytokines that are secreted by these activated cells [72,74,75]. In the initial stages of PS, myeloid dendritic cells (mDCs), activated by key cytokines such as TNF-α, INF-α and INF-y, and IL-6, migrate into the draining lymph nodes and secrete mediators such as IL-12 and IL-23, driving differentiation and proliferation of Th-1 lymphocytes and Th-17, respectively. These T cells, in turn, produce a wide variety of cytokines such as IL-17, IL-21, IL 22, IL-26, TNF-α, and INF-γ, which induce the hyperproliferation of keratinocytes with a consequent increase in the thickness of the epidermis and the production of antimicrobial peptides, chemokines, and proinflammatory cytokines, contributing to the amplification of psoriatic inflammation (see Figure 1). Therefore, the unmodulated action of these inflammatory molecules secreted by immune and resident cells plays a crucial role in the development and persistence of inflammation in psoriatic lesions [76,77,78].

SOCS proteins, as reported above, are present in various tissues including the skin and are known to act as classical negative feedback inhibitors. In particular, these molecules are produced in response to different cytokines and, in turn, suppress their signaling in order to balance immune responses [40]. Various studies have shown that SOCS proteins modulate the activity of several cytokines in PS through cellular signaling pathways. In the pathogenesis of PS, INF-γ, secreted by infiltrating T lymphocytes, is a potent activator of keratinocytes that, following stimulation, produce many proinflammatory cytokines [79]. The results of this in vitro study showed that human keratinocytes, transiently transfected with SOCS1 gene and co-transfected with the IFN-y-inducible reporter plasmid, overexpressing SOCS1, were resistant to the inflammatory activity of IFN-γ [79]. This resistance resulted in inhibition of IFN-γ-induced phosphorylation of IFN-yRα, STAT1, and STAT3. Furthermore, SOCS1 also reduced IFN-γ-induced production of chemokines and adhesion molecules, including ICAM-1, HLA-DR, IFN-γ-inducible protein-10 (IP-10), and monocyte chemoattractant protein-1 (MCP-1). The authors of this work suggest that SOCS1 could be a potential molecular target for the treatment of IFN-γ-induced immune-mediated skin diseases such as PS [79]. Moreover, Doti and colleagues identified PS-5, a mimetic peptide of the KIR region of SOCS1, by screening a focused peptide library [80]. They then tested this peptide in human keratinocyte cultures and demonstrated that PS-5 reduced IFN-γ-induced STAT1 phosphorylation [80]. In addition, in a psoriasis-like experimental inflammatory model, it was reported that PS-5 suppressed IFN-γRα and impaired phosphorylation of JAK2 and STAT1α in IFN-γ-activated human keratinocytes or human skin explants treated with PS-5. Immunohistochemical analysis, moreover, highlighted that PS-5 reduced the expression of proinflammatory genes such as ICAM-1, HLA-DR, CXCL10, and CCL2 [81]. The same authors demonstrated, in an in vitro cell contact model, a reduced migratory capacity of T lymphocytes towards keratinocytes pretreated with PS-5 and stimulated with IFN-γ [81]. These data suggest that the SOCS1 mimetic peptide has therapeutic potential in the treatment of immune-mediated inflammatory skin diseases such as PS. It is known, in addition, that in the pathogenesis of PS, IFN-α and T lymphocytes are also involved in the infiltration of the epidermis. In this respect, it was demonstrated that CD8+ T cells from psoriatic lesions, in response to IFN-α, showed reduced expression of SOCS3 and a low baseline expression of the SH2-domain-containing protein tyrosine phosphatase (SHP)-1 compared to T cells from healthy donators in response to IFN-α. Moreover, SOCS3-deficient T cells showed marked upregulation of STAT1 activation in the presence of IFN-α. These data argue that there is a loss of regulatory control of IFN-α signaling in PS [82].

Other authors found that specific deletion of SOCS3 in mouse keratinocytes caused severe skin inflammation. The inflamed skin showed hyperactivation of constitutive STAT3 and overexpression of IL-6. Upregulation of IL-6 induced increased expression of IL-20-receptor-related proinflammatory cytokines (IL-20R) such as IL-19, IL-20, and IL-24 which, in turn, increased hyperactivation of STAT3 and hyperproliferation of keratinocytes. In these transgenic mice, disruption of the balance between IL-6, STAT3, and SOCS3 induced a severe clinical phenotype of psoriatic-like keratinocytes [83]. It was also demonstrated that, in mice, deletion of SOCS3 in keratinocytes using the K5-Cre transgene resulted in STAT3 hyperactivation, epidermal hyperplasia, and impaired healing of skin wounds, so the authors of this work argued that keratinocytes play an essential role in the control of skin wounds through the gp130–SOCS3–STAT3 pathway [84]. It was reported, moreover, that SOCS-3 deficiency leads to sustained activation of STAT3 in response to IL-6, which is a cytokine abundantly present in psoriatic plaques [85,86]. Immunohistochemical analysis highlighted an overexpression of the SOCS3 protein in keratinocytes of the basal layer in healthy skin and a suppression of the protein in the epidermis of psoriatic plaques. This suppression was related to an upregulation of miRNA-203 in psoriatic lesions. The authors hypothesized that downregulation of SOCS3 in psoriatic keratinocytes results in prolonged STAT3 signaling activity induced by inflammatory cytokines, leading to immune cell infiltration and the development of psoriatic lesions [87]. In addition, an immunohistochemical study performed on skin biopsies of psoriatic patients revealed an elevated expression of the molecules STAT1, STAT3, SOCS1, SOCS3, VEGF, and PAK1 in keratinocytes and in the dermis compared to the control group [22]. The same study also observed an mRNA overexpression of all six JAK/STAT and VEGF/PAK signaling molecules in psoriatic skin compared to healthy skin. Such findings suggested that these signaling molecules could provide topical therapeutic targets for this disease [22]. Conversely, Madonna and colleagues found that overexpression of SOCS1 and SOCS3 proteins in human psoriatic keratinocytes suppresses apoptosis of these cells in response to IFN-γ and TNF-α and that keratinocyte survival is activated by the anti-apoptotic PI3K/AKT pathway [88]. Furthermore, histological analysis of the skin of these psoriatic patients revealed peculiar epidermal thickening following upregulation of both SOCS1 and SOCS3 [88] and that the increased expression of SOCS1 levels in keratinocytes of psoriasis patients and activated by IFN-γ is not sufficient to counteract the detrimental effects of this cytokine [89]. It was also observed that the overexpression of SOCS3 in the keratinocytes of transgenic mice results in aggravation of the inflammatory wound [90].

Moreover, a recent study conducted in patients affected by PS has shown that SOCS3 and SOCS7 polymorphism seems have no influence on psoriasis; however, a limitation of the study is the small sample size taken into consideration, so further studies are needed to understand the impact that SOCS genes have on psoriatic disease [91].

**Figure 2:**
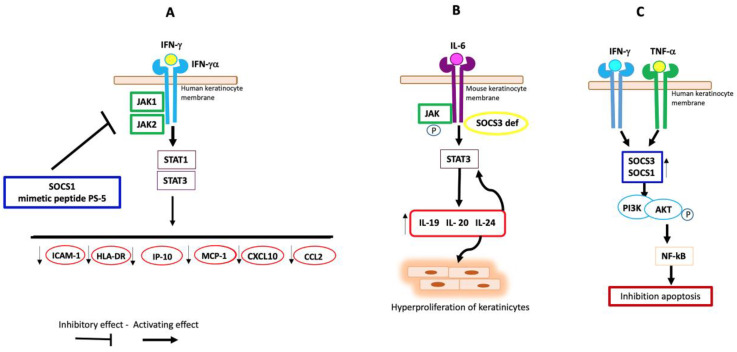
Possible involvement of SOCS proteins in PS. (**A**) In human keratinocytes, SOCS1 and its mimetic peptide PS-5 reduce the production of ICAM-1, HLA-DR, IP-10, MCP-1, CXCL10, and CCL2 through inhibition of JAK1/2 and the consequent downregulation of STAT1/3, blocking IFN-γRα-induced signal. (**B**) Deletion of SOCS3 in mouse keratinocytes induces increased expression of IL-20R-receptor-related proinflammatory cytokines and hyperactivation of STAT3 in response to IL-6. (**C**) In human psoriatic keratinocytes, the cytokines IFN-g and TNF-a induce high levels of SOCS1 and SOCS3 expression, which activates PI3K with consequent phosphorylation of AKT. The latter determines the phosphorylation of the transcription factor NF-κB, which induces the gene expression of anti-apoptotic molecules.

Taken together, these data indicate that SOCS1 and SOCS3 proteins may protect against the harmful and prolonged activity of inflammatory cytokines through JAK/STAT signaling in inflammatory skin diseases (Figure 2). The results also suggest that modulating the overexpression of SOCS may have greater efficacy in the protective action of these molecules. Further studies are needed to fully understand the therapeutic efficacy of SOCS in psoriasis.

### 4.2. SOCS Proteins in AD

AD, also called atopic eczema, is the most common chronic inflammatory skin disease and mainly affects young children but also adults with an increasing incidence in more developed countries. It has been indicated by evidence from several experimental and clinical studies that other forms of atopic disorders such as allergic rhinitis, asthma, and food allergies can often develop in AD patients [92,93]. Typical signs of skin inflammation of this pathological condition include itchy and painful patches, severe edema, erythema, dryness, scaling, and scabbing, but these vary from patient to patient and can affect small or larger areas of skin. These manifestations, especially the persistent itching, also cause profound functional disorders, such as disturbances in sleeping, inability to carry out normal activities of daily life, and psychosocial distress [94,95,96]. The pathogenesis of this atopic disorder is complex, and understanding it is complicated due to the involvement of different components that act synergistically to influence the manifestations of AD. AD pathogenesis involves genetic, environmental, and immunological factors that interact with each other, causing an impairment of the structure and function of the skin barrier, an imbalance of the skin microbiome, and a dysfunction of the innate and adaptive immune system [97,98]. The dysfunction of the skin barrier is associated with a reduction in the structural protein filaggrin, epidermal lipids, and antimicrobial peptides and a dysbiosis of commensal bacteria, which together determine a loss of epidermal integrity, skin dehydration, chronic inflammation of the skin, and increased sensitization to allergens and infections (see Figure 1) [99,100].

A damaged epithelial barrier therefore leads to an accumulation of proinflammatory cytokines and chemokines secreted by activated innate immune cells, including proinflammatory mediators released by keratinocytes. This inflammatory cascade induces a progressive infiltration of different types of inflammatory cells. The acute phase of the disease is characterized by an early epidermal infiltration of CD4+ Th2 cells that secrete high levels of various cytokines such as IL-4, IL-5, IL-13, and IL-31. Subsequently, there is an accumulation of mature dendritic cells, macrophages, eosinophils, and mast cells which are responsible for the secretion of a mixed pattern of Th2/Th1 cytokines and chemokines that promote the chronic inflammation typical of AD. In addition, during the development of the disease, other subsets of lymphocytes come into play such as Th22 and Th17 cells [101,102]. Th22 cells release IL-22, which induces epidermal hyperplasia and inhibits terminal differentiation of keratinocytes, and while the role of Th17 cells is controversial, IL-17 infiltration into atopic lesions has been shown to modify keratinocytes to induce proinflammatory mediators [103,104,105] (see Figure 1).

Dysregulation of immune mediators including cytokines could be one of the key elements involved in the inflammation of AD.

The SOCS protein family, well known as negative regulators of cytokine signaling by interfering with the binding of cytokine receptors and the intracellular molecules that act downstream, such as JAKs and STATs, could be involved also in modulating the JAK/STAT pathway in AD and related diseases such as allergic rhinitis, asthma, and food allergies [23,106,107]. These negative regulators influence the skin inflammatory process at multiple stages, such as the development and differentiation of Th2 cells and the related production of proinflammatory cytokines, or by maintaining a balance between the cytokines that regulate Th1- and Th2-mediated immune responses [108,109,110]. It has been observed that SOCS molecules are differentially expressed in Th1 and Th2 phenotypes. Th2 cells expressing high levels of SOCS3 repress IL-12-induced STAT4 activation while IL-4/STAT6 signaling is inhibited by the Th1 phenotype exhibiting high SOCS1 expression. These proteins can be considered markers of the Th lineage and can serve as therapeutic targets for immune modulation therapy [111].

Federici et al. have demonstrated that SOCS1, SOCS2, and SOCS3 proteins were highly expressed in the keratinocytes of patients affected by allergic contact dermatitis but poorly expressed in those with atopic dermatitis. In both diseases, leukocytes infiltrating the dermis showed immunoreactivities for all three regulatory molecules [79]. In contrast, histological analysis has highlighted a marked expression of both SOCS3 mRNA and protein in skin samples from patients with atopic dermatitis, suggesting that SOCS3 has a significant role in AD [112]. Others reported that elevated levels of SOCS3 in T cells promote Th2 and reduce Th1 differentiation in patients with AD and asthma, leading to an increased risk of these Th2-mediated pathologies. Marked expression of SOCS3 induces IL-12-mediated inhibition of Th1 cell differentiation, enhancing Th2 cell development in transgenic mice [113,114]. In addition, the transgenic mice constitutively expressing SOCS3 showed an increase in Th2 cell responses. In the dominant-negative mutant SOCS3 in transgenic mice but also in mice with heterozygous deletion of SOCS3, there was reduced differentiation of Th2 cells [113]. These results suggest that SOCS3 may have an important role in Th cell differentiation and activation.

Data from a cDNA microarray study showed that SOCS3 mRNA is markedly expressed in the skin of patients with AD compared to healthy skin and that specific haplotypes of SOCS3 are strongly associated with AD in childhood cohorts [115]. In addition, immunohistological analyses highlighted increased SOCS3 protein levels in dendritic cells and an increased number of SOCS3-positive cells in the AD skin epidermis. These finding suggest that the SOCS3 protein could be an important target for innovative therapeutic protocols [115]. Conversely, in the peripheral blood of AD patients, SOCS3 mRNA levels were elevated compared to those of healthy controls, and these levels were unchanged after the application of effective therapy, suggesting that SOCS3 is not associated with skin lesions but with maintaining the Th2-dominant phenotype in AD [116]. Studies have shown that IL-6 promotes Th2 cell responses and inhibits Th1 cell ones via IL-4 expression induction in allergic inflammatory conditions [117,118]. SOCS3 negatively regulates IL-6-mediated JAK/STAT signaling [119].

In vivo studies have shown that silencing of SOCS3 leads to an exacerbation of allergic inflammation [120]. Furthermore, the increased expression of SOCS3, highlighted in allergic patients, suppresses the increased activity of IL-6 and STAT3. This implies that the SOCS3 protein has a protective action in various allergic inflammatory conditions [121]. Other studies, on the other hand, reported that in atopic dermatitis, including AD, overexpression of SOCS3 was associated with increased serum levels of IL-6 compared to control cases, suggesting a protective role of the SOCS3 protein [113,116]. The 174-promoter region of the IL-6 gene has been reported to contain a functional polymorphism that modifies serum IL-6 levels [122].

In addition, a study conducted on the polymorphism of the IL-6 promoter region (IL-6 174-G/C) in patients affected by AD reported that genotypes with a protective effect presented high levels of SOCS3, which were associated with the presence of high levels of IL-6 detected in the serum, so the authors suggest that SOCS3 together with IL-6 could be potential indicators of diagnosis/prognosis in atopic diseases including AD [121,122]. Studies have observed that SOCS5 regulates naïve T cell differentiation by dampening IL-4 signaling [121,123]. In Th1 cells, the SOCS5 protein is preferentially expressed and inhibits Th2 differentiation through IL-4 via STAT6 [45], but disease severity does not appear to correlate with SOCS5 in patients affected by AD [116]. Therefore, the SOCS5 protein could play a role during differentiation of Th/1Th2 phenotype lymphocytes. In an allergic asthma model, high levels of SOCS5 in transgenic mice resulted in increased Th2 responses and eosinophilic inflammation, suggesting that SOCS5 overexpression in Th2-dominated diseases is not a useful therapeutic target [124]. It was reported that mice lacking SOCS7 developed AD-like manifestations with skin thickening and injuries, and histological examination showed that the epidermis of these mice had an increase in the number of mast cells and of granular mast cells compared to wild-type litter mates. The same result was obtained in healthy mice lacking SOCS7.

In addition, SOCS 6/7 double knockout mice showed more severe skin inflammation, suggesting that the SOCS7 protein plays a regulatory role in mast cells [125]. In an in vivo model of AD, SOCS2-deficient mice had increased susceptibility to AD. Histological analysis displayed more severe skin excoriations accompanied by epidermal hyperplasia, a marked infiltration of eosinophils and dermal T cells compared to wild mice. These mice also showed increased Th2 responses and decreased IL-17 levels compared to wild-type mice. This implies that the SOCS2 protein could have an inhibitory effect on atopic responses. In addition, SOCS2-deficient CD4^+^ T cells showed constitutively increased levels of STAT6 phosphorylation compared to wild-type T cells, suggesting that these T cells lacking SOCS2 were more responsive to IL-4 [126].

Moreover, in children with AD, it has been reported that serum levels of SOCS3 and STAT3 proteins were higher than in controls. Furthermore, STAT3 but not SOCS3 levels had a very positive correlation with the SCORAD index of childhood AD. The authors of these observations hypothesize a translocation of the two proteins in the extracellular space through the plasma membrane.

To date, there are no literature data that describe the mechanisms by which these proteins translocate into the extracellular fluid and the related biological function.

The marked levels of SOCS3 and STAT3 in the serum of children with AD suggest that these two proteins can be used as biomarkers to reflect the severity of the disease; therefore, their therapeutic modulation could be important in preventing the development of the disease [127].

**Figure 3:**
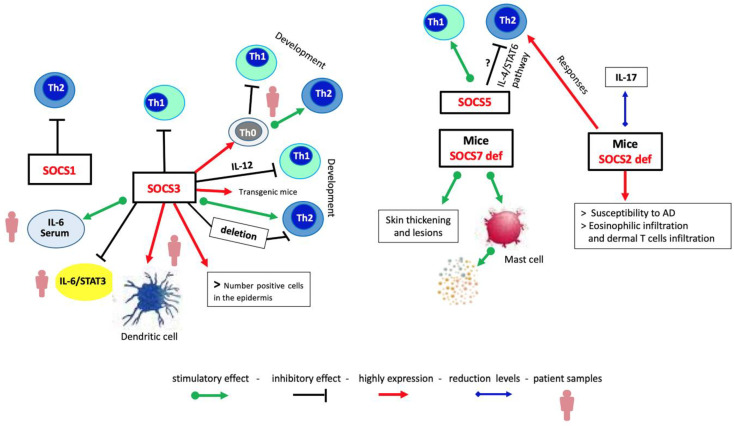
Mechanistic view of immune response regulation by SOCS proteins in AD. SOCS proteins are differently expressed in Th cell populations. High expression of SOCS1 leads to the Th1 responses, whereas SOCS3 drives the Th2 responses; SOCS5 promotes the Th1 responses, reducing those of Th2 cells probably through the IL-4/STAT6 pathway. SOCS7 deletion develops AD-like manifestations and an increase in the number of granular mast cells. Deletion of SOCS2 protein induces the appearance of AD-like signals.

From these data, it emerges that some members of the SOCS protein family have, in various ways, a significant role in atopic diseases including AD (Figure 3). Therefore, the activities of these negative regulators could be useful as therapeutical targets in atopic diseases.

### 4.3. SOCS Proteins and Wound Healing

Skin wound healing is a complex and dynamic process that restores the integrity of skin after an injury. It consists of four highly orchestrated overlapping phases known as hemostasis, inflammation, proliferation, and remodeling. The inflammatory phase involves the recruitment of white blood cells to the wound site with the aim to clean up debris and protect against infection [128].

The proliferation phase is characterized by the formation of new blood vessels (angiogenesis) and the production of new tissue. During this phase, fibroblasts lay down collagen, and epithelial cells start to cover the wound surface. After this phase, during the remodeling process, collagen fibers are reorganized so the wound contracts, and the new tissue gains strength [128].

Due to their involvement in the regulation of the inflammatory response as a crucial cellular process for wound healing, SOCS proteins have been explored as potential therapeutic targets for wound healing, but the results of various studies have not always revealed a unique role for these proteins.

SOCS proteins can help to prevent excessive inflammation, which can damage tissues and delay healing. They can also promote the growth and differentiation of new skin cells. In this regard, it was reported that different SOCS proteins may have distinct roles in wound healing. For example, SOCS3 has been shown to inhibit the proliferation and migration of keratinocytes, which are essential for skin repair, suggesting a role in regulating the timing and extent of these processes involved in would healing [55]. On the other hand, it has been also demonstrated that the exacerbated inflammation that characterizes chronic wounds could be associated with the overexpression of SOCS3 [90]. However, it was also shown that deletion of SOCS3 in mouse keratinocytes resulted in hyperactivation of STAT3, epidermal hyperplasia, and impaired skin wound healing [84]. These observations are evidence of a different role for SOCS protein in regulating both inflammatory events and skin repair processes.

In addition, the results of a study by Behm and colleagues suggested that SOCS1 promotes inflammatory cell infiltration, leading to a prolonged inflammation phase, a major cause of chronic wounds [129].

Conversely, other authors report that both SOCS1 and SOCS5 modulate IL-4 signaling to regulate re-epithelialization and the tissue-remodeling phase of wound healing [130]. Moreover, SOCS3 and SOCS4 are reported to play regulatory roles both in keratinocyte and endothelial cellular traits associated with the wound-healing process and that they may also be able to regulate the responsiveness of these cells to EGF and TGFβ, thus implying a potential regulatory role in skin repair [131].

Overall, the role of SOCS proteins in wound healing is multifunctional and depends on the specific type of SOCS protein apart from the stage of the healing process. Studies on the specific roles of SOCS proteins in wound healing are currently limited and not fully elucidated. Further research is needed to help to understand how SOCS proteins can be targeted to improve wound-healing outcomes.

### 4.4. SOCS Family Proteins Aging, Photoaging, and Senescence-Associated Secretory Phenotype (SASP)

SOCS proteins and photoaging are part of an emerging field of research, although there is still limited evidence directly linking SOCS proteins to photoaging.

Photoaging refers to the premature aging of skin caused by chronic exposure to sun UV radiation characterized by wrinkles, fine lines, age spots, and loss of skin elasticity [132]. Recent studies focus on the regulatory role that SOCS proteins play in the immune system by suppressing the activity of cytokines, which are signaling molecules also involved in aging skin [133]. In this respect, it was reported that UVB radiation suppresses the expression of SOCS3, which could lead to increased inflammation and contribute to skin aging [134].

SOCS proteins, therefore, might regulate the inflammatory response caused by UV exposure, acting on signaling pathways essential for maintaining skin health, but the potential link between SOCS proteins and photoaging is still in the early stages of investigation, and further research is necessary to fully clarify the underlying mechanisms.

SASP is a crucial aspect of cellular senescence, a state of permanent cell cycle arrest triggered by various stressors. Although beneficial in suppressing tumor formation, SASP can also exhibit detrimental effects due to its proinflammatory nature [135,136]. When skin cells become senescent, reaching a state of growth arrest, they shown a secretory phenotype, called SASP, that encompasses proinflammatory cytokines, matrix-degrading enzymes, including matrix metalloproteinases, and dysregulated growth factors [137]. This altered secretome perpetuates chronic low-grade inflammation and compromises the extracellular matrix, contributing significantly to the structural and functional hallmarks of cutaneous aging [138].

In this respect, SOCS proteins could act as negative regulators of SASP, limiting its proinflammatory effects. Dysregulated SOCS proteins might allow for heightened and uncontrolled inflammatory signaling, amplifying the SASP and worsening its aging effects on skin. This, however, remains only a hypothesis because there is still no direct evidence about this aspect, since studies on the specific roles of SOCS proteins in aging, photoaging, and SAPS are very limited and incomplete. Elucidating the SOCS proteins’ involvement, their mechanisms of dysregulation, and their downstream effects on functional aspects of SASP may represent a promising target to identify novel therapeutic strategies in the management of both photoaging and intrinsic skin aging.

## 5. Conclusions

During the onset and progression of inflammatory skin diseases such as PS and AD, the local microenvironment is characterized by a persistent presence of inflammatory mediators, such as cytokines, that induce keratinocytes and both resident and infiltrating immune cells, amplifying immune responses with deleterious consequences for the skin environment. Regulation of the signaling of these inflammatory cytokines by SOCS proteins appears to be essential for the regular progression, regression, and remodulation of the immune response in order to pursue protective action. From the data in this review, it emerges that members of the SOCS protein family, as physiological intracellular negative regulators of cytokine signaling, could be considered as valid candidates in the treatment of inflammatory skin diseases to suppress the harmful, unsteady, and prolonged signaling of inflammatory cytokines through the modulation of the JAK/STAT signal pathway. Therefore, further studies are needed to explore the function of SOCS proteins in order to understand the role of the balance between the different members of the SOCS protein family in the pathogenesis of these skin inflammatory diseases for quicker and more effective therapeutic treatment of these disorders.
